# Pharmspresso: a text mining tool for extraction of pharmacogenomic concepts and relationships from full text

**DOI:** 10.1186/1471-2105-10-S2-S6

**Published:** 2009-02-05

**Authors:** Yael Garten, Russ B Altman

**Affiliations:** 1 Biomedical Informatics Training Program, Stanford University, Stanford, CA, USA; 2 Departments of Bioengineering and Genetics, Stanford University, Stanford, CA, USA

## Abstract

**Background:**

Pharmacogenomics studies the relationship between genetic variation and the variation in drug response phenotypes. The field is rapidly gaining importance: it promises drugs targeted to particular subpopulations based on genetic background. The pharmacogenomics literature has expanded rapidly, but is dispersed in many journals. It is challenging, therefore, to identify important associations between drugs and molecular entities – particularly genes and gene variants, and thus these critical connections are often lost. Text mining techniques can allow us to convert the free-style text to a computable, searchable format in which pharmacogenomic concepts (such as genes, drugs, polymorphisms, and diseases) are identified, and important links between these concepts are recorded. Availability of full text articles as input into text mining engines is key, as literature abstracts often do not contain sufficient information to identify these pharmacogenomic associations.

**Results:**

Thus, building on a tool called Textpresso, we have created the Pharmspresso tool to assist in identifying important pharmacogenomic facts in full text articles. Pharmspresso parses text to find references to human genes, polymorphisms, drugs and diseases and their relationships. It presents these as a series of marked-up text fragments, in which key concepts are visually highlighted. To evaluate Pharmspresso, we used a gold standard of 45 human-curated articles. Pharmspresso identified 78%, 61%, and 74% of target gene, polymorphism, and drug concepts, respectively.

**Conclusion:**

Pharmspresso is a text analysis tool that extracts pharmacogenomic concepts from the literature automatically and thus captures our current understanding of gene-drug interactions in a computable form. We have made Pharmspresso available at .

## Background

To catalyze progress in understanding the molecular basis of drug response and its variability in humans, pharmacogenomics researchers need to establish connections between genes, gene polymorphisms, drugs, and diseases. However, pharmacogenomics is a dynamic field with a growing literature, and this wealth of published information can be difficult to track because it is published in many discipline-specific journals. To address this difficulty, researchers sometimes perform multiple searches repetitively – differing only in their syntax but not semantics – to find facts that match certain patterns. For example, a query template may be "{gene} VERB {drug}", where the {gene} and {drug} are a specific gene and drug of interest to the researcher, and the verb phrase is one of the following three: 'binds', 'interacts with', or 'associates with'. These searches are very similar, and yet must be performed separately to extract all articles that contain each of the particular triplets, as there is no existing method to search using a category that includes the three verb phrases. Text mining approaches may be useful in addressing this problem, because computational techniques can automatically scan, retrieve and summarize the literature and store it in a computable format. Search techniques such as PubMed and Google are keyword-based, and do not contain semantic information about desired relationships in text. Natural language processing has been applied to pharmacogenomics in the past [1,2], but there is an opportunity now to use it for extracting the connections between the entities of interest in pharmacogenomics. Template-based semantic search can allow these connections to be automatically extracted, building on the commonalities in the sentence structure. The templates must be tailored to the specific field of research, in order to incorporate the terms and categories of interest to the researcher.

Separate from the technical issues of information extraction is the choice of text corpus. A major limitation of many search methods is that only literature abstracts are indexed. However, the full text of the articles may offer improved performance.

Some systems can support semantic concept-based search or relationship extraction, including Relemed [3], iHOP [4], Ingenuity Pathways [5], GENIES [6], CBioC [7], and GeneWays [8], but these systems do not generally provide search within full text and visual mark-up of the search results within the local context.

The Textpresso search engine is a template-based text search engine developed for Model Organism databases [9]. Textpresso uses an expert-built ontology that contains categories of phrases and words of biological interest. Database curators and users specify particular types of objects and relationships of interest, and the system finds articles that match these. Textpresso is based on a large set of regular expressions written to find templated relationships in text. It indexes the full text of articles that are provided as PDF files. Constraints can be added to queries – for example, specifying that two entities appear in the same sentence in the article. Highlighted search results allow the searcher to efficiently skim search results.

We hypothesized that with minor modifications Textpresso would be useful for the task of identifying and extracting pharmacogenomic relationships. In particular, we wanted to extend Textpresso to be useful for pharmacogenomics literature focusing on drugs, human genes, their variants, and the associated molecular and cellular phenotypes. Articles processed by our modified Textpresso (Pharmspresso) are selected to be relevant to pharmacogenomics based on previously reported tools [1,10]. Table 1 shows examples of categories and terms identified by Pharmspresso. We evaluated our extension by comparing the ability of Pharmspresso to extract information about genes, drugs and polymorphisms from 45 articles, to the performance of 11 human gold standard evaluators reading the same literature.

**Table 1:** Pharmspresso ontology examples.

**CATEGORY TYPE**	
**Biological entity**	**Example words/phrases**

Cell or cell group	germ line cells, intenstines, sensory neurons
Cellular component*	axons, integrins, mitochondrial membrane
**Disease**	stroke, chronic leukemia, tuberculoma
**Drug**	acebutolol, mechlorethamine, tartaric acid
**Gene**	ABCB1, CYP2C9, coagulation factor V
Organism	mice, rat, xenopus laevis
**Polymorphism**	T168N, 1039G-A, 236Arg->Lys



**Relationships between entities**	**Example words/phrases**

Action	assists, acomplishes, recognizes
Association	associates, binds, interacts
Biological Process*	acetylated, matures, reactivations
Characterization	has, contains, displays, includes, lacks
Comparison	correlates, differs, equally, matches
Effect	accumulates, aggregates, causes

Examples of Textpresso biological entities and relationships, along with additions for Pharmspresso (in bold). The ontology includes 35 categories of two types: (1) biological entities and (2) relationships between entities. Category names and examples of these categories are shown. * marks categories imported from Gene Ontology (GO).

## Results

### Pharmspresso overview

Table 2 gives an overview of the Pharmspresso database. The current corpus contains 1025 full text articles from 343 different journals. Future releases of Pharmspresso will incorporate larger numbers of articles, as well as abstracts in cases where full text was not available.

**Table 2:** Overview of the Pharmspresso database.

# articles	1,025
# journals	343
# gene terms recognized*	102,334
# drug terms recognized	3,756
# disease terms recognized**	36,843

* Includes names, symbols, aliases

** Includes redundancies in MeSH thesaurus

We used the MeSH thesaurus disease terms, including many synonyms and phrase permutations that create redundancy in disease matches. However, these are required to capture the different ways in which they appear in natural language.

**Figure 1:**
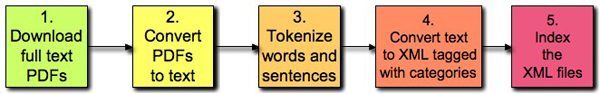
**Pharmspresso pipeline for data processing**. The Pharmspresso pipeline for data processing: full text PDFs of articles are downloaded, converted to text, and tokenized into individual words and sentences. Next, the text is parsed to identify words or phrases that are members of specific categories within the ontology. These are marked as such and indexed for future search accessibility.

Figure 1 shows the Pharmspresso pipeline for document retrieval and information extraction. We were able to significantly improve runtime of the markup algorithm, which can now mark up 1000 articles in less than 5 minutes on a single core consumer grade PC.

### Pharmspresso can extract facts relevant to a specific gene

**Figure 2:**
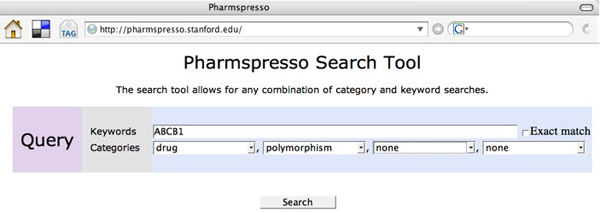
**Pharmspresso search page**. Snapshot of Pharmspresso search page. User is searching for text that includes the keyword 'ABCB1' as well as a member of the {drug} category and a member of the {polymorphism} category, within the abstract or full text.

**Figure 3:**
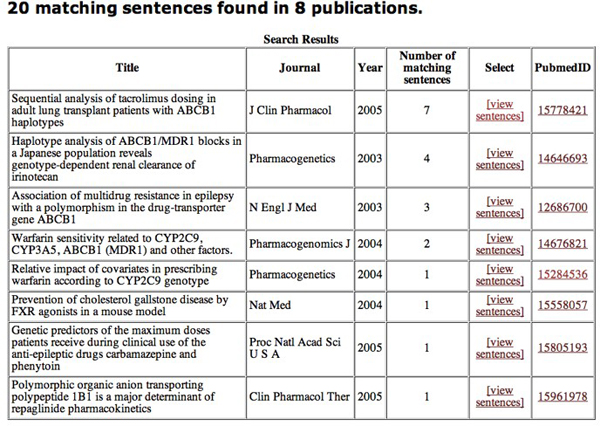
**Pharmspresso results page**. Results page for the search shown in Figure 2. There are eight publications (from the corpus of 1025 in Pharmspresso) that include a total of 20 sentences fulfilling the query conditions. Users may view the sentences in each of these articles that match the query. The number of matches indicates the number of sentences containing the query keywords and categories.

Figure 2 shows a snapshot of the Pharmspresso search page. It shows a typical scenario in which users want articles establishing a relationship between a drug and a gene variant. Users can specify keywords and categories from the ontology that should appear within the same sentence in an article. The search results show all articles fitting these criteria, as shown in Figure 3. In the displayed query, the user searched for all articles that mention ABCB1 (which is a gene), along with any term from the drug category and a polymorphism. The snapshot shows that the corpus contains 8 articles that contain such a sentence, with a total of 20 such sentences.

**Figure 4:**
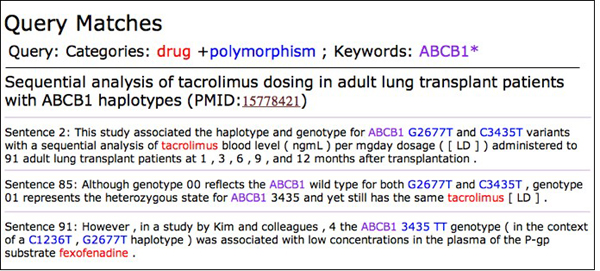
**Marked-up sentences found in corpus which match user query**. Sentences matching the query are color-coded with keywords and categories highlighted. In this example, 'tacrolimus' is a member of the {drug} category, and 'G2677T' and 'C3435T' are members of the {polymorphism} category. Pharmspresso displays the title and sentence number within the text.

Figure 4 shows the specific sentences found by the search engine, color-coded based on the query, allowing swift perusal of search results. Several sentences that matched the query are displayed; the first hit is the only one that is part of an abstract, and would thus be available on PubMed.

### Pharmspresso can extract categorical facts and relationships between categories of biological entities

In other queries, particular relationships of interest can be specified in addition to keywords. For example, a user may require a relationship synonymous with "association" or "effect" to be found within a sentence. In particular, patterns such as "{drug} {association} {gene}" can be found by querying for sentences containing these three categories. Specific instantiations of {gene}, such as 'CYP2C19', can be sought by querying for the keyword 'CYP2C19' with the categories {drug} and {association}. For example, the article by Ha-Duong et al. titled 'Ticlopidine as a selective mechanism-based inhibitor of human cytochrome P450 2C19', includes the following sentence retrieved by Pharmspresso: "Spectral interaction studies demonstrate that **ticlopidine **readily **binds **to the protein active site of **CYP2C19**" [11].

### Pharmspresso finds information not present in abstracts alone

**Figure 5:**
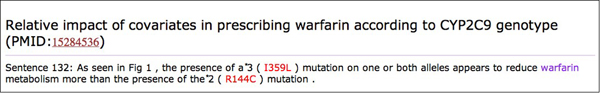
**Pharmspresso retrieves sentences from full text not found when scanning abstract only**. User queried for 'warfarin' keyword + a member of the {polymorphism} category. Results show that the article titled 'Relative impact of covariates in prescribing warfarin according to CYP2C9 genotype' contains such a sentence, but this sentence would not be found by reading abstract only, as it is sentence number 132 in the article, which actually appears in the 'Discussion' section. Although the 'star notation' (*2, *3) is used earlier in the article to describe gene variants, explicit genomic location information which can be used to map this polymorphism is first given in sentence 132.

**Figure 6:**
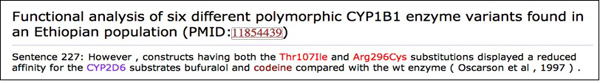
**Pharmspresso retrieves fact from referenced article**. User queried for both keywords 'CYP2D6' and 'codeine' and a member of the {polymorphism} category. Although the article ('Functional Analysis of Six Different Polymorphic CYP1B1 Enzyme Variants Found in an Ethiopian Population') discusses the gene 'cytochrome P450 1B1' and not 2D6, there is a reference to knowledge in a referenced article, regarding a polymorphism in CYP2D6 (not in CYP1B1) and its affect on affinity for codeine. Thus, this article is extracted in response to the query.

Figure 5 shows the result of a query that finds no relevant sentences from the article abstract, but does find a sentence in the full text. The importance of full text in information extraction has been detailed in [9]. Figure 6 shows another example of sentences found in the full text but not in the abstract. The fact retrieved is a terse summary of findings from a different article. In this case, the referenced article is not in the Pharmspresso corpus, but the summary in full text is nevertheless still retrieved.

### Evaluation of Pharmspresso system

We performed an evaluation of the system by comparing the information extraction by Pharmspresso to a gold standard of manual curation (see Methods for details). The 45 articles used in our evaluation contain 178 gene mentions; Pharmspresso finds 78.1% (139) of these. The gold standard contains 255 polymorphism mentions; Pharmspresso finds 48.6% (124). When compared to the non-table gold standard (see Methods for description of this), Pharmspresso performance rises to 60.8% [124/(255-51)], as there are 51 mentions that appear in tables embedded in the article as images. The gold standard contains 191 drug mentions; Pharmspresso finds 74.4% (142). If we query {gene} and {drug} together, Pharmspresso finds 50.3% of these associations. Table 3 contains a summary of these results.

**Table 3:** Pharmspresso performance in evaluation.

**Entity Type**	**% Recovered**
Gene	78.1
Polymorphism	48.6
Polymorphism (non-table gold standard)	60.8
Drug	74.4

Summary of Pharmspresso performance in evaluation. The rows report percent of gene, gene-variant (polymorphism), and drug mentions found by the gold standard, recovered by Pharmspresso.

## Discussion

Pharmspresso's main strengths are its ability to process full text articles and index their contents based on an ontology of key concepts, on a corpus of literature relevant to the field of pharmacogenomics. It provides a search engine for finding entities and semantic relationships between them of pharmacogenomic importance. Pharmspresso identifies relationships because it has a model of the words used to associate different concepts. Its display allows users quickly to browse through search results.

Because Pharmspresso uses regular expressions that may be imprecise, it can retrieve false positive search results. For example, in one article (PMID 15564882) the ligand 'E1S' is tagged as a polymorphism, similar in pattern to E216S which indicates a substitution at position 216 from glutamic acid to serine. Fortunately, the highlighted search results are easy to peruse, and irrelevant hits can quickly be discarded by users. The parsing of author names in the reference section of an article also leads to false positive gene polymorphisms, and the bibliographies should probably be removed from the corpus.

Some instances are missed by regular expressions that are too precise. For example, the gene name OCT-1 appears in the literature, whereas our lexicon contains the more standard notation OCT1 for this gene. Pharmspresso misses OCT-1 as a gene. Careful refinement of gene name templates could improve performance.

The creators of the Textpresso ontology often included the category name as a term in the category, such as the term 'cell' in the category 'cell or cell group'. This practice was useful to us, in particular with the inclusion of the word 'gene' in the {gene} category. Pharmspresso can thus highlight gene mentions, even if the gene itself does not appear in our lexicon; we can identify genes because the word 'gene' is highlighted. For example, in the sentence from PMID 12123487: 'the individual response to gemfibrozil could be partly explained by polymorphisms in genes coding for apolipoproteinB (apoB) and apoA1CIII', the drug gemfibrozil is reported to be in a relationship with the genes 'apolipoproteinB' and 'apoA1CIII', which do not appear in the Pharmspresso drug lexicon. However, because the word 'genes' does appear in the lexicon, this text snippet is highlighted, with the word 'genes' highlighted, and thus allows the researcher to examine the article in which the sentence appears. This also allows us to refine our gene lexicon to include the two genes mentioned. Similarly, if we expand the polymorphism category to identify the words 'polymorphism', 'variant', and 'allele', performance is likely to improve.

Our focus on the identification of polymorphism mentions in the literature, was in identifying those variants that can be mapped to specific locations in the genome. Thus, Pharmspresso does not recognize gene variant names that follow the "star notation" (such as 'CYP2A6*4') as a polymorphism, as they do not give any explicit genomic location information. However, Pharmspresso can easily be engineered to identify these variants, perhaps using a new category in the ontology. For example, in the 45 articles reviewed by the evaluators, there were 117 mentions of variants that follow the star notation. Pharmspresso uses a relatively simple algorithm for finding polymorphisms. Others have reported more complex methods – sometimes more domain-specific and usually more computationally expensive for finding gene and variant mentions [12-17]. These may be incorporated in future versions of our system.

In our evaluation of polymorphism and gene detection, one of the 45 articles contained a large table with all the mentions of polymorphisms. However, as this table was an image embedded in the article, the conversion from PDF to text did not capture this information. This is a limitation of the system. For the purposes of this analysis, that article was removed and the analysis of polymorphism mentions included only 44 articles. We find that the most important polymorphisms are often also mentioned in the text of the article itself, and thus are not missed by Pharmspresso.

We note that there were some *bona fide *polymorphisms in the literature that the annotators missed – these would have raised performance in identification of polymorphism mentions.

Pharmspresso identifies 74.4% of drug mentions when querying for the {drug} category, as opposed to only 50.3% if querying for {gene} and {drug} categories together. Often, the gene is described in once sentence, and the drug in the following sentence. Thus the strict limit of one sentence only might best be relaxed to allow 2 or more sentences. Pharmspresso does not currently have a mechanism to rank the most likely or most frequently mentioned associations. Such a ranking could assist users in deciding which associations are the most reliable.

A bottleneck in the process of the Pharmspresso pipeline is the gathering of the full-text articles. It can be tedious to download PDFs from journals that publish work on Pharmacogenomics. We anticipate that the improved availability of full text may permit partnerships with publishers to streamline the pipeline. Another limitation of the system is that Pharmspresso only works on a pre-defined corpus of relevant articles, and not on all existing literature. In the future, Pharmspresso will include a larger corpus, and abstracts will be used when the full text is not available. Additionally, the results of the information extraction will be downloadable in tabular format, useful for building interaction networks.

The Pharmspresso package is available to the public at .

## Conclusion

Pharmspresso is a resource that extracts information from full text articles by identifying key pharmacogenomic concepts and the relationships between them. It marks up a corpus of literature based on an ontology of concepts, among which are the classes genes, gene variants, drugs, and polymorphisms. Subsequently, it displays the sentence-level results to the user as visually enhanced text, highlighting the relevant extracted concepts within their local context. Pharmspresso is easily extendible to other disciplines. As the increasing amount of published literature makes it very difficult for humans to manage the knowledge in a scientific field, automated tools are needed to organize the information and effectively understand unstructured text. We are working on making Pharmspresso a robust system that will automatically retrieve relevant literature, mark it up using our ontology, extract the facts of interest, and use them to populate a database of interactions. In addition to serving as a resource for human users, the knowledge collected by Pharmspresso may also be amenable to automated data mining and relationship-discovery methods.

## Methods

### Pharmspresso ontology

We created the Pharmspresso ontology by adding to the existing Textpresso ontology the human gene, drug, polymorphism and disease categories. The gene list uses names, symbols and aliases described by the HUGO Gene Nomenclature Committee (HGNC [18]), as well as additional gene names found in the literature and compiled by the team of PharmGKB scientific curators. The drug list was created by using the PharmGKB drug dictionary, which includes drug lists compiled by Apelon Inc. and Micromedex, as well as manual entries. Polymorphisms are recognized by regular expressions written in the Perl programming language, which include the common ways in which polymorphisms are described in the literature (e.g. A3435T, ARG23LYS, and variations), as well as rs and ss numbers corresponding to dbSNP entries [19]. The disease category includes disease terms of the MeSH thesaurus, as well as additional disease names found in the literature and compiled by the team of PharmGKB scientific curators.

### Pharmspresso implementation

We used the Textpresso open source package to build Pharmspresso. A corpus of literature was downloaded from the web, totaling 1025 full text articles in PDF format. We used a free software package, xpdf , to convert PDF files to text; Perl scripts adapted from Textpresso were used to tokenize sentences and words. We implemented a tagging algorithm in Perl to tag text with the Pharmspresso ontology in XML format, and subsequently index the tagged text for use by the search engine.

CGI scripts access the database and create the HTML pages shown to the user. Figure 1 shows the Pharmspresso pipeline. In step 4 of the pipeline, each article is parsed for identification of all terms in the ontology that appear in the text. This process takes less than five minutes per 1000 full text articles on a single core consumer grade PC. Steps 2, 3 and 5 take a total of several minutes of computational time for the entire corpus.

We built a website for Pharmspresso, which allows search of the current corpus of literature, and is available at .

### Pharmspresso corpus

The corpus of literature included in the current build of Pharmspresso (1025 downloaded full text PDFs) is papers currently in the PharmGKB, which have been manually verified as relevant to pharmacogenomics. We can also identify relevant literature by automatic extraction using a text classifier [1,20], by receiving submissions from the user community, or via annotation by PharmGKB curators.

### Evaluation

We evaluated Pharmspresso as follows: (1) We recruited gold standard evaluators (scientists familiar with pharmacogenetics literature), and sent each five articles (selected from the PharmGKB annotated corpus), with instructions to find within the full text of the article all mentions of genes, polymorphisms (with the associated gene name), and drugs. (2) Eleven evaluators reviewed a total of 45 articles. (3) We measured (i) the percent of gold-standard gene-mentions found by Pharmspresso when querying for sentences containing a term from the {gene} category, (ii) the percent of polymorphism mentions found by Pharmspresso when querying for sentences containing terms from both the {polymorphism} and {gene} categories, and (iii) the percent of drug mentions found by Pharmspresso when querying for sentences containing a term from the {drug} category.

When evaluating the performance of Pharmspresso in identification of polymorphism mentions, we compared to two versions of the gold standard: (i) the strict gold standard, which includes all mentions found by the evaluators, and (ii) a "no-table" gold standard, which removes from consideration those mentions annotated by the evaluators, that appeared only in a table embedded in the PDF as an image (and thus were not converted to text, thereby impossible for Pharmspresso to detect).

## Competing interests

The authors declare that they have no competing interests.

## Authors' contributions

YG wrote the software, created the website, and analyzed the data. YG and RBA together conceived the project and wrote the manuscript.
